# *Rhododendron aureum* Georgi formed a special soil microbial community and competed with above‐ground plants on the tundra of the Changbai Mountain, China

**DOI:** 10.1002/ece3.3307

**Published:** 2017-08-11

**Authors:** Xiaolong Wang, Lin Li, Wei Zhao, Jiaxin Zhao, Xia Chen

**Affiliations:** ^1^ National and Local United Engineering Laboratory for Chinese Herbal Medicine Breeding and Cultivation School of Life Sciences Jilin University Changchun China

**Keywords:** function microbial, interspecific competition, microbial biomass, microbial community, *Rhododendron aureum*, soil microbial

## Abstract

*Rhododendron aureum* Georgi is a perennial evergreen dwarf shrub that grows at all elevations within the alpine tundra of northern China. Previous research has investigated the plant communities of *R. aureum*; however, little information is available regarding interspecific competition and underground soil microbial community composition. The objective of our study was to determine whether the presence of *R. aureum* creates a unique soil microbiome and to investigate the relationship between *R. aureum* and other plant species. Our study site ranged from 1,800 to 2,600 m above sea level on the northern slope of the Changbai Mountain. The results show that the soil from sites with an *R. aureum* community had a higher abundance of nitrogen‐fixing bacteria and a higher resistance to pathogens than soils from sites without *R. aureum*. We emphasize that *R. aureum* promotes a unique soil microbial community structure that is distinct from those associated with other plants. Elevation and microbial biomass were the main influencing factors for plant community structure. Analysis of interspecific relationships reveals that *R. aureum* is negatively associated with most other dominant shrubs and herbs, suggesting interspecific competition. It is necessary to focus on other dominant species if protection and restoration of the *R. aureum* competition is to occur. In the future, more is needed to prove whether *R. aureum* decreases species diversity in the tundra ecosystems of Changbai Mountain.

## 1 INTRODUCTION

Soil microbes play a key role in ecosystems and mediate many ecological processes that are critical to decomposition, nutrient cycling, and plant community dynamics(Fierer et al., 2012; Van der Putten, Klironomos, & Wardle, 2007). In addition, they can act as a source and sink for plant nutrients (Nannipieri et al., 2012). The interaction between plants and soil microbes is at the forefront of international ecological research (Mendes et al., 2011; Park et al., 2006). Studies have shown that different plant species host‐specific microbial communities, suggesting that plant species play an important role in shaping the soil microbiome (Berendsen, Pieterse, & Bakker, 2012; Oh et al., 2012). Microbial biomass and activity are typically thought to be constrained by soil nutrients (Liu et al., 2015). Studies reveal that plant production and diversity increases the soil microbial biomass by altering the environmental factors, for instance by increasing the concentration of nitrogen and organic matter (Eisenhauer et al., 2013; Lange et al., 2015). Furthermore, the microbial community composition of soil has a large impact on plant–plant interactions (Bever, Platt, & Morton, 2012) and, consequently, on plant diversity and composition (Heijden et al., 2006; Schlatter, Bakker, Bradeen, & Kinkel, 2015).

So far, there were many researches have reported about the shifting in microbial community structure and functional genes along a large‐scale alpine climosequence (Collins, Carey, Aronson, Kopp, & Diez, 2016; Cui et al., 2016; Zhang, Liang, He, & Zhang, 2013). At relatively small scales, individual plants can also alter the composition of the soil community (Bever et al., 2010; Putten et al., 2013); however, there is currently a relatively limited understanding of how different plants affect the microbial community structure and function in alpine tundra that was not formed with a vertical distribution of vegetation.

In the alpine environment, interspecies relationships within the community are important for plants. Many studies have confirmed widespread positive interactions (facilitation) within alpine plant communities (Baumeister & Callaway, 2006; Schob et al., 2014). Previous research has also demonstrated the existence of competition or “parasitic” interactions between benefactor and beneficiary species in alpine ecosystems (Kikvidze et al., 2005; Maestre, Callaway, Valladares, & Lortie, 2009; Schob et al., 2014). Moreover, soil microbes have a substantial impact on plant productivity, community composition, and diversity (van der Heijden, Bardgett, & van Straalen, 2008). Because plants cannot fix atmospheric nitrogen, which is a main limiting factor for their growth, nitrogen‐fixing bacteria are important regulators of plant productivity (Iii, 2003). Soil microbes can decompose soluble and insoluble organic matter and convert it into organic and plant‐effective forms (Raab, Lipson, & Monson, 1999). Accelerated carbon and nitrogen cycling, as well as decomposition in the soil, stimulate microbial activity, thereby enhancing shrub development (Bret‐Harte et al., 2001; Hobbie, Nadelhoffer, & Högberg, 2002; Naito & Cairns, 2011; Sturm et al., 2005; Syndonia, Shaver, & Stuart, 2002).

Alpine tundra of the Changbai Mountain is one of two rare alpine tundra ecosystems in China (Zhao, Qi, Lyu, Yu, & Chen, 2016). *Rhododendron aureum* Georgi is a perennial evergreen shrub found in Northeast Asia that is able to endure the cold alpine climate (Kudo, 1993; Zhao et al., 2016). It is the only woody plant distributed at all elevations throughout the tundra and it plays an important role in maintaining the ecological balance, while preventing and controlling soil erosion. These unique plant communities can produce specific physical and chemical properties within the soil through their litter and root exudates, thus influencing the soil microorganisms (Cheng et al., 2015). Past studies have focused on the plant communities (Jin et al., 2015; Liu et al., 2012; Zhao et al., 2016), with little research into interspecific competition and underground soil community composition.

*Rhododendron aureum* is highly adaptive and can thrive at various altitudes. It is crucial to understand the complexity of this adaptive processes and the main forces driving population structure (Joost et al., 2007; Mosca, Gonzálezmartínez, & Neale, 2014). In this study, we (1) hypothesize that on a small‐scale, *R. aureum* influences the soil microbial community, distinct from other species; (2) explore the relationship between *R. aureum* and other species in alpine tundra; (3) discuss the interaction between plant community structure and soil properties. To test our hypothesis, we set an altitudinal gradient ranging from 1,800 m to 2,600 m above sea level using a systematic sampling design. We established pairwise treatments at the same elevation, consisting of sites with *R. aureum* and sites with other species but without *R. aureum*. We then analyzed the soil from both treatment types with regards to soil properties, N‐cycling, microbial function, and microbial community composition. Finally, we evaluated the diversity variation within the native above‐ground communities and predicted the biotic interactions.

## 2 MATERIALS AND METHODS

### 2.1 Study area

**Figure 1:**
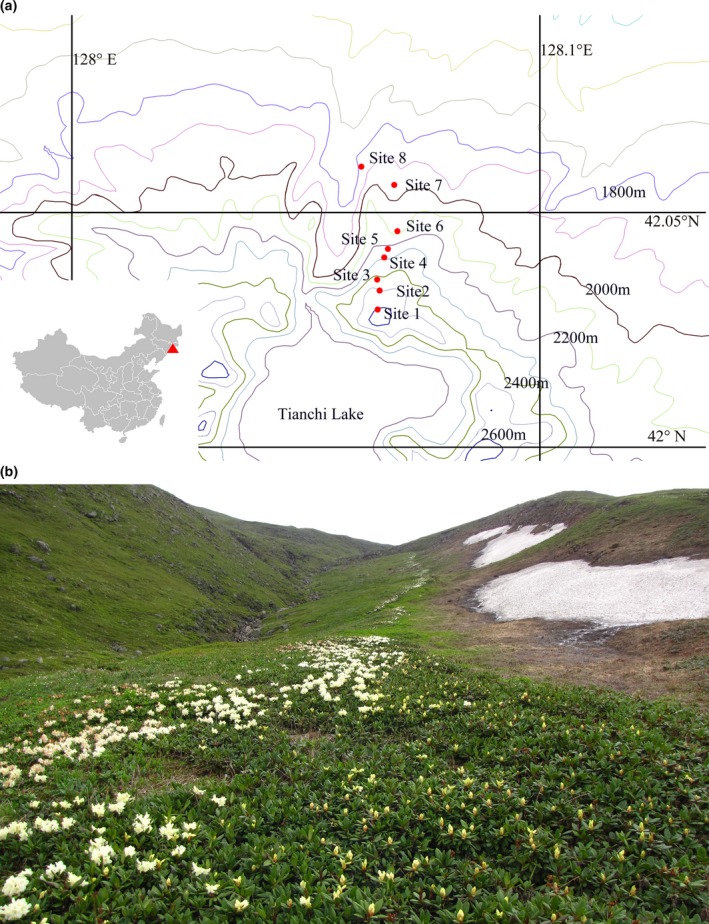
(a) Distribution of eight sites sampled along an elevation gradient on the tundra of Changbai Mountain. We extracted the elevational information from DIVA‐GIS Free Spatial Data (http://www.diva-gis.org/Data) and created the map by the program Global Mapper version 13.00 (http://www.bluemarblegeo.com/products/global-mapper.php) and DIVA‐GIS version 7.5.0 (b) The population status of *R. aureum* in site 4 from Changbai Mountain

Sampling sites were established on the northern slope of the Changbai Mountain which is acknowledged as the highest mountain in northeastern China and eastern Eurasia. The Changbai Mountain has clearly defined vertical vegetation zones: deciduous broad‐leaved forest, mixed deciduous broad‐leaved/conifer forest, dark coniferous forest, *Betula ermine* forest, and tundra (Zheng, Wallin, & Hao, 1997). The study area was located on the northern slope at elevations from 1,800 m to 2,600 m above sea level (Figure 1). The climate is characterized by low temperatures, heavy precipitation, and a short‐growing season. The average annual temperature is 3–7°C, with an annual precipitation is over 600 mm (Yang & Wu, 1998). In accordance with the alpine climate, common plants are of low stature or prostrate shrubs, with *R. aureum* being the most dominant evergreen shrub in the study area. Other plants grow here include *Dryas octopetala*,* Vacciniumu liginosum L*., *Ligularia jamesii (Hemsl.) Kom, Trollius chinensis, Rhodiola cretinii,* and *Sanguisorba tenuifolia var*. (Yang & Wu, 1998; Zong et al., 2014).

### 2.2 Experimental design and sampling

Fieldwork was conducted from 2014 to 2015 along an altitudinal gradient ranging from 1,800 to 2,600 m, using a systematic sampling design. A total of 16 plots from eight sample sites were established in different altitudinal sites covering all the dominant species associated with *R. aureum* in the alpine ecosystem of Changbai Mountain. In each site, we established plots consisting of at least 90% *R. aureum* and <10% non‐*R. aureum* species (with *R. aureum*; N1‐N8) and plots with 100% non‐*R. aureum* species (without *R. aureum*; X1–X8). Each plot measured 20 m × 20 m and contained 12 shrub subplots, measuring 5 m × 5 m, and 12 herb subplots, measuring 1 m × 1 m. The foliage coverage of vascular plant species in each plot was recorded as percentages, along with their height, by visual estimation of each species.

Soil samples were collected in mid‐July 2015 at the 16 established plots. At each plot, 12 soil cores at depths of 0–10 cm were collected, using a core sampler with a 10 cm inner diameter at a distance of 8 m around the plot center. The 12 cores were pooled to obtain one composite sample for each plot and immediately stored on ice in insulated containers. After returning to the laboratory, each sample was processed by passing it through 2‐mm mesh and dividing it into two groups: one group was stored at 4°C for chemical analyses and the other one at −80°C for microbial community analysis.

### 2.3 Analysis of soil characteristics

Soil moisture was determined after drying at 105°C; pH was determined by mixing soil and deionized water at a 1:5 mass to volume ratio. Soil total nitrogen (TN) and alkaline hydrolysis nitrogen (AN) were measured by the semimicro‐Kjeldahl (KDY‐9820) digestion and alkali diffusion method, respectively (Bao, 2000). Available N (ammonium and nitrate) was extracted with 2 mol/L KCl and then measured by indophenol‐blue and phenoldisulfonic acid colorimetry, respectively (Bao, 2000). Soil total phosphorus (TP) and total potassium (TK) were determined by the molybdate method (Murphy & Riley, 1962) and extracted by incubation with sodium hydroxide, respectively. Available phosphorus (AP) was determined by the Olsen method (Olsen et al., 1954), and available potassium (AK) was determined using the ammonium acetate extraction procedure (Bao, 2000). Microbial biomass, C and N (MBC and MBN), was extracted with 0.5 mol/L K_2_SO_4_ and analyzed using the chloroform fumigation extraction method (Brookes, Landman, Pruden, & Jenkinson, 1985). Phosphatase activity was determined with the modified Schinner and von Mersi method (Schinner & Mersi, 1990). Catalase activity and invertase activity were determined using the 0.1 N KMnO_4_ titration method (Johnson & Temple, 1964) and the 3,5‐dinitrosalicylic acid technique (Vaughan & Ord, 1980), respectively. Urease activity was determined according to Klose and Tabatabai (Klose & Tabatabai, 2000).

### 2.4 Soil DNA extraction

DNA was extracted from 0.3 g of freeze‐dried soil using the Power Soil DNA isolation kit (MoBio). Extracted DNA was purified using the GV‐High‐Efficiency Agarose Gel DNA Purification Kit. DNA concentrations were checked with the Qubit quantification platform. DNA was diluted to 10 ng/μl using TE buffer and then stored at −80°C prior to molecular analysis.

The V3‐V4 hypervariable region of the bacterial 16S rRNA gene was amplified using the primers U341F (5′‐ACT CCT ACG GGA GGC AGC AG‐3′) and U806R (5′‐GGA CTA CHV GGG TWT CTA AT‐3′). An equal amount of PCR product for each sample, as measured by Qubit quantification platform, was pooled in a single tube to be sequenced using the Illumina MiSeq platform (Realbio Genomics Institute, Shanghai, China). The raw data were then subjected to quality control using UPARSE (Edgar, 2013). The sequences were quality‐filtered and clustered into operational taxonomic units (OTUs) at a 97% similarity level using USEARCH (Edgar, 2010). A representative sequence for each OTU was assigned to a taxonomic level using the RDP (Ribosomal Database Project) database by the RDP classifier (Maidak et al., 1996).

The relative abundance of genes encoding key enzymes for biological N‐cycling (i.e., *nifH*, archaeal amoA, bacterial amoA, and *nosZ*) was assessed by qPCR (ABI 7500). The gene fragments were amplified using the primers PolF/PolR for *nifH*,* Arch‐amoA*F/*Arch‐amoA*R for archaeal amoA, *amoA*‐1F/*amoA*‐1R for bacterial amoA, and *nosZ*‐F/*nosZ*‐1622R for *nosZ*(Levy‐Booth, Prescott, & Grayston, 2014; Mao, Yannarell, Davis, & Mackie, 2013). Primers and qPCR conditions are described in Table S1. Standards for the qPCR assays were generated with PCR amplicons from the pooled DNA sample (Chen, Yu, Jr, Wittum, & Morrison, 2007). The copy number of the genes in each standard was calculated by dividing the DNA concentration (ng/μl) by the average molecular weight of the amplified gene fragment (Mao et al., 2013). qPCR amplification efficiencies ranged from ~90% to 110%, with *R* ^2^ values between 0.992 and 0.999.

### 2.5 Statistical analysis

The coverage (%) of plant communities was transformed to an ordinal scale and assigned to one of four coverage classes according to the 1–4 Lagerberg–Raunkiaer scale (Ludwig & Reynolds, 1988) and used for the interspecies association analysis (Guozhen Du & Wang, 1989). The importance value of a species, which is considered the most realistic and important element in assessing vegetation inventory, is useful in comparing the ecological significance of species in a given area (Curtis & McIntosh, 1951; Lamprecht, 1989). This value was calculated from the sum of the relative density, relative frequency, and relative dominance, as recommended by Kent and Coker (1992). The species diversity was determined using the Shannon–Wiener diversity index (H’) and Pielou index (Pielou, 1969).

General linear model analysis (mean ± *SD*,* n* = 5) of variance was performed to test differences between sites with and without *R. aureum*. Tukey's LSD post hoc test was used to identify significant differences. Statistical analyses were performed using SPSS (v. 19.0 for Windows, SPSS Inc., Chicago).

The number of OTUs in a community was used to estimate its richness and structure. Alpha diversity was measured by the Chao1 and Shannon indices. Beta diversity of the microbial communities was investigated using multivariate analysis and nonmetric multidimensional scaling (NMDS). A Bray–Curtis distance matrix was calculated using the soil microbial OTU data using the vegan package in R statistical software (v.3.1.1; Oksanen, Kindt, Legendre, & O'Hara, 2010). Treatment differences among these metrics were assessed using analysis of similarities (ANOSIM) in R. Additionally, redundancy analysis (RDA) was performed, which is an extension of principal components analysis, where the main components are constrained to linear combinations of environmental variables. To determine the environmental factors that significantly correlate with community composition (abundance of OTUs or relative abundance of species), the envfit function (999 permutations) in the vegan package of R was used.

## 3 RESULTS

### 3.1 Soil nutrient dynamics

**Figure 2:**
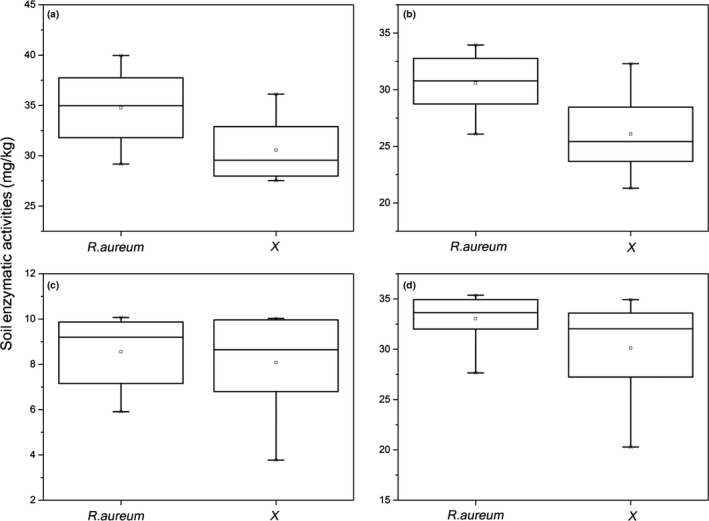
Soil enzyme activities difference between the two treatments sites (with and without *R. aureum*). (a): Urease, *p* = .03; (b): Sucrase, *p* = .015; (c): Acid phosphatase, *p* = .637; (d) Catalase, *p* = .165. X indicates the sites without *R. aureum*

The thirteen soil parameters varied across the two treatment sites (with and without *R. aureum*; Table 1). Sites with *R. aureum* were generally more nutrient rich in terms of nutrient pools (TN, TOC, TP, and TK), moisture, and soil pH; however, these values were not significantly different when compared to the sites without *R. aureum* (*p *>* *.05). Only microbial biomass carbon and nitrogen (MBC and MBC) were significantly higher (*p *<* *.05) than sites without *R. aureum*. To investigate the cause of these changes, we performed correlation analysis between soil properties and elevation (Table S2). Out of nine parameters were significantly correlated with elevation (Table S2). Among them, TK and pH positively correlated with elevation, while other parameters negatively correlated. Furthermore, four soil enzymatic activities were measured (Figure 2). When comparing the two treatment types, sites with *R. aureum* contained a higher amount of enzymatic activity. Specifically, sucrase and urease were significantly higher in sites with *R. aureum* (*p *<* *.05, Figure 2).

**Table 1:** Soil variables (mean ± *SD*) were from two treatments

	TN	AN	TP	AP	TK	AK	NO_3_^‐^	NH_4_^+^	TOC	pH	MBC	MBN	Moisture
*R. aureum*	7.86	474.82	561.60	12.86	15.22	135.84	33.98	123.67	145.02	4.81	1514.95	401.20	50.38
other species (X)	6.70	336.75	429.65	12.27	14.34	143.44	40.77	129.39	117.78	4.77	1,338.63	352.09	40.81
*p* value	.34	.21	.18	.90	.61	.89	.64	.85	.32	.77	**.00**	**.01**	.26

Each elevation has eight sampling points. Bolded values indicate contrasts that are significantly different (*p* < .05) between different treatments.

**Figure 3:**
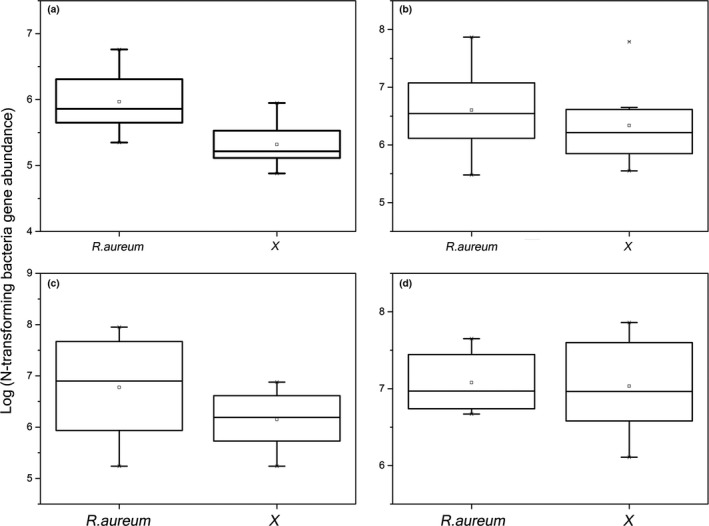
Real‐time PCR quantification of the N‐cycling function genes (*nifH*, archaeal *amoA*, bacterial *amoA,* and *nosZ*) in the bulk soil under two treatments. The copy number of genes in each gram of dry soil was estimated based on the results of real‐time PCR (copies per ng DNA). (a): *nifH, p* = .007; (b): bacterial *amoA*,* p* = .483; (c): archaeal *amoA, p* = .149; (d) *nosZ, p* = .865. X indicates the sites without *R. aureum*

The abundance of N‐cycling genes was not significantly different (*p *>* *.05) between treatment sites (Figure 3), with the exception of the bacterial N‐fixing gene nifH. The abundance of nifH was higher in sites with *R. aureum* than without (*p *<* *.05; Figure 3). Correlation analysis showed that the abundance of N‐cycling genes was significantly correlated (*p *<* *.05) with some soil parameters (Table 2). Abundance of the nifH gene was positively correlated with total nutrients (TOC and TN), available nutrients (AN and AP), microbial biomass (MBC and MBN), and soil moisture (Table 2). The abundance of AOA (archaeal amoA) was negatively correlated with soil nitrate nitrogen (NO_3_^−^); however, the abundance of N‐cycling genes was not significantly correlated with ammonium nitrogen (NH_4_^+^) and AK. The abundance of AOB (bacterial amoA) was negatively correlated with AK, but positively correlated with soil pH and elevation; however, abundance of other N‐cycling genes was not significantly correlated with elevation. The abundance of nosZ was positively correlated with TP and AP (Table 2).

**Table 2:** Relationships between the abundance of N‐cycling function genes and soil properties

	Altitude	TN	AN	TP	AP	TK	AK	NO_3_^−^	NH_4_^+^	TOC	pH	MBC	MBN	Moisture
nifH														
*r*	−.42	**.58**	**.65**	.46	**.49**	−.40	.48	−.20	−.11	**.55**	−.45	**.70**	**.57**	**.53**
*p* value	.10	**.02**	**.01**	.07	**.05**	.12	.06	.46	.69	**.03**	.08	**.00**	**.02**	**.03**
AOA														
*r*	−.12	.00	.13	−.14	−.30	.00	−**.49**	−**.55**	−.17	.20	.01	.33	.26	.30
*p* value	.67	.99	.63	.61	.26	1.00	**.05**	**.03**	.54	.45	.98	.21	.34	.26
AOB														
*r*	**.78**	−.49	−.47	−.07	−.43	.41	−**.65**	.11	.25	−.38	**.50**	−.23	−.34	−.45
*p* value	**.00**	.06	.07	.81	.10	.11	**.01**	.68	.36	.15	**.05**	.40	.19	.08
nosZ														
*r*	.10	.34	.15	**.50**	**.56**	.00	.44	.27	.36	.01	−.33	.03	−.01	.03
*p* value	.71	.20	.58	**.05**	**.03**	.99	.09	.30	.17	.98	.22	.93	.96	.90

The value is correlations (*r*) and *p* value. The Bolded values indicate contrasts that are significantly different (*p* ≤ .05) between different treatments.

### 3.2 Soil microbial communities

**Figure 4:**
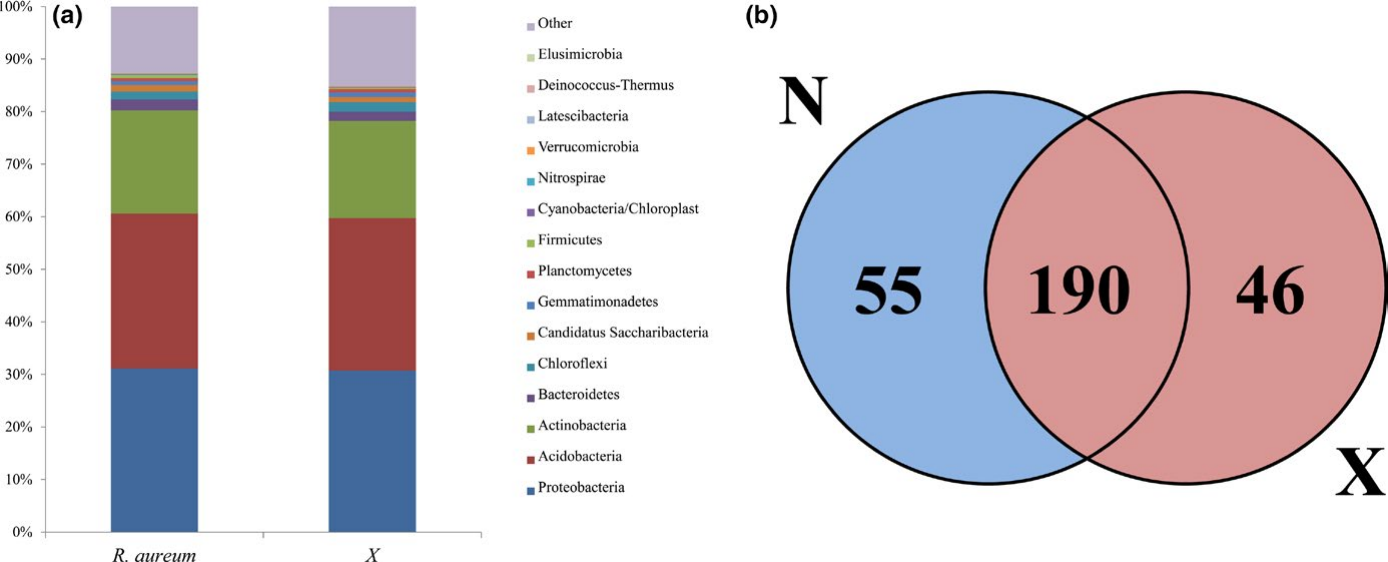
(a) Relative abundances of the dominant bacterial phyla in soils separated according to elevation and species categories. Relative abundances are based on the proportional frequencies of those DNA sequences that could be classified at the phylum level. (b) Venn diagrams representing and the counts of reads from unique to and shared between the different treatments. N indicates *R. aureum* and X indicates the treatment of other species without *R. aureum*

Sequencing of the 16S rRNA gene resulted in 431,560 sequences from 16 samples following the removal of low‐quality sequences, chimeras, and denoising. The number of sequences per sample ranged from 19,009 to 31,653, with an average sequence count of 26,973. These sequences represented 2,022 OTUs at 97% similarity. The bacterial community composition was relatively consistent between soil samples from sites with and without *R. aureum*. At the phylum level, the majority of sequences corresponded to Acidobacteria (30.6%), Proteobacteria (29.6%), and Actinobacteria (18.7%; Figure 4a). Species annotation revealed that *R. aureum‐*associated soil contained 245 bacterial genera and soil from sites without *R. aureum* contained 236 bacterial genera, with 190 shared genera between them (Figure 4b).

**Figure 5:**
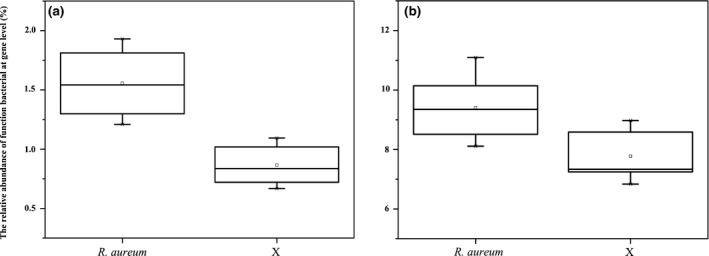
Relative abundances of the dominant function bacterial at gene level. (a) Pathogenic properties bacterial, *p *<* *.001; (b) nitrogen fixation bacteria, *p* = .04

Alpha diversity analysis shows that the microbial diversity of soils with *R. aureum* was lower than that of soil samples without *R. aureum*, but not significantly (Table S3); however, the relative abundance of genera associated with nitrogen fixation and pathogenesis were significantly higher within *R. aureum*‐associated soil (Figure 5).

**Figure 6:**
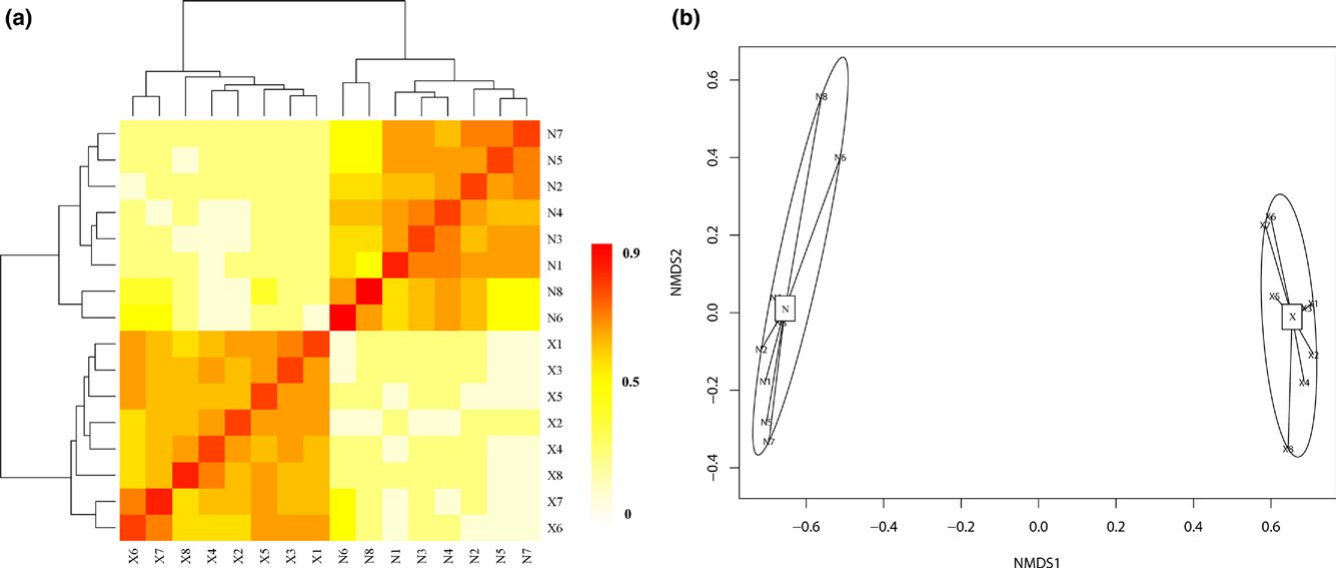
(a) Pairwise comparison of β‐diversity with all samples and annotations. Clustering and heatmap were computed using R package. (b) Nonmetric multidimensional scaling (NMDS) plot of community composition based on pyrosequencing. N indicates *R. aureum*, X indicates the treatment of other species without *R. aureum*

**Figure 7:**
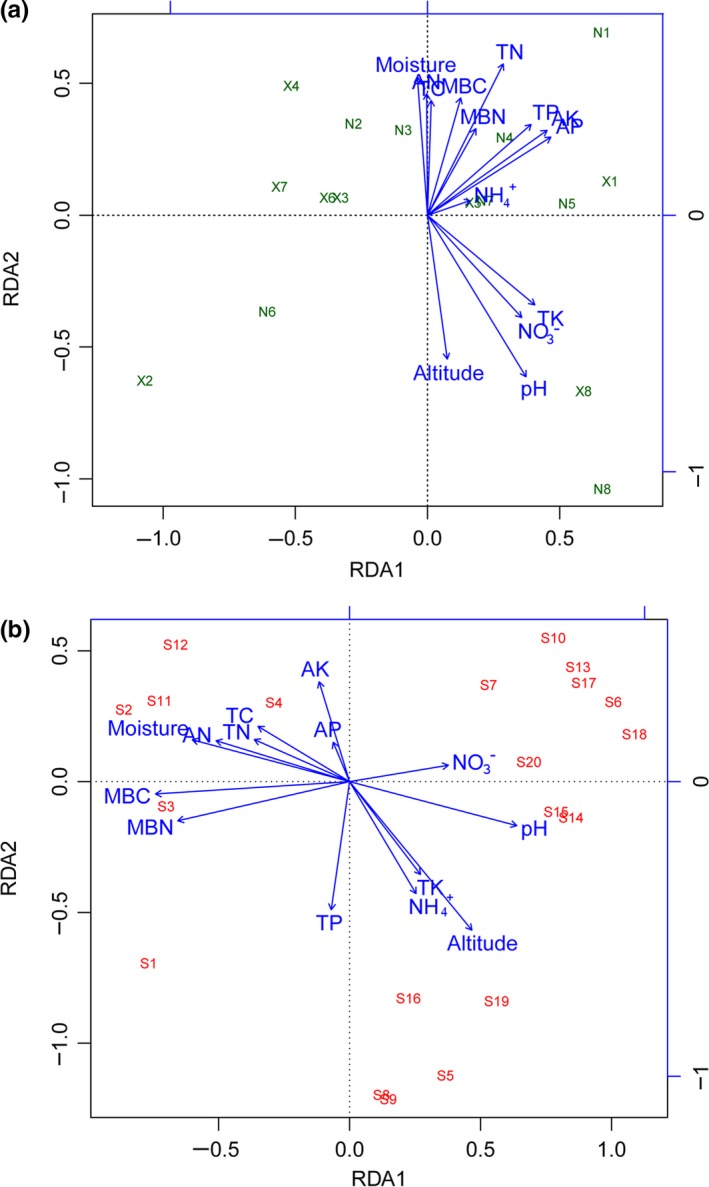
(a) Redundancy analysis (RDA) triplots of 16S rDNA fingerprint patterns, showing the contribution of 14 environmental parameters to variability. (b) Redundancy analysis (RDA) triplots of plant species richness showing the contribution of 14 environmental parameters to variability. Arrows indicate environmental factors and their relative effects on microbial community structure. S1, *R. aureum*; S2, *Vaccinium vitis*‐*idaea*; S3, *Juniperus sibirica*; S4, *Vaccinium Spp*; S5, *Salix divaricata var*. *meta*‐*formosa*; S6, *Dryas octopetala var*. *asiatica*; S7, *R*. *confertissimum*; S8, *R. bracteatum*; S9, *Phyllodoce caerulea*; S10, *Saussurea tomentosa*; S11, *Streptopus koreanus*; S12, *Deyeuxia angustifolia*; S13, *Oxytropis anertii*; S14, *Polygonum viviparum*; S15, Gen*t*iana *algida*; S16, *Anthoxanthum monticola*; S17, *Dendranthema oreastrum*; S18, *Lloydia serotina*; S19, *Tofieldia coccinea*; S20, *Festu caovina*

The heatmap shows pairwise comparisons of bacterial community structures between all samples (Figure 6a). Results indicate that the sites with *R. aureum* cluster into one clade, while the sites without *R. aureum* cluster into another. NMDS ordination also reveals that microbial communities were significantly different between soils with and without *R. aureum* (*p *=* *.01; Figure 6b). In order to better understand the influence of soil microbial communities, we investigated the interdependence between 16S rDNA transcript abundance of individual phylotypes and single environmental factors using RDA (Figure 7a). Of all the environmental variables tested, TN (*r* ^*2*^ = 0.43, *p* = .02) and pH (*r* ^*2*^ = 0.54, *p* = .03) demonstrated the highest correlation with community composition (Table S4). Other factors, such as elevation (*r* ^*2*^ = 0.32, *p* = .09) and AP (*r* ^*2*^ = 0.32, *p* = .09) also showed a high correlation with bacterial community composition (Table S4). The abundance of Gemmatimonadetes (*r* = .686, *p* = .003)*,* Bacteroidete*s* (*r* = .706, *p* = .002), and Nitrospirae (*r* = .694, *p* = .003) was positively correlated with soil pH. TN was negatively correlated with Gemmatimonadetes (*r* = −.541, *p* = .03) and positively correlated with Fusobacteria (*r* = .776, *p* = .001; Fig. S1).

### 3.3 Interspecific relationships

Within the 16 sampling plots, a total of 81 plant species were encountered, belonging to 31 families and 58 genera. Based on the overall importance values, a relative measure accounting for the sum and frequency of coverage, the most important species were *R. aureum, Vaccinium uliginosum, Saussurea tomentosa*, and *Streptopus koreanus* Ohwi. The vast majority (77%) of the species had importance values below 1%, indicating that they were locally rare. Variance analysis shows that the overall interspecific correlation of the nine dominant shrubs was significantly positive, (VR = 1.55, W = 148.73, χ^2^ (0.95)96 = 74.4), and that the distribution of the species was not independent; however, when using individuals to assess the abundance index, there is a significant negative association between *R. aureum* and other accompanying species at the 99% confidence level (Table 3).

**Table 3:** Interspecies association between *Rhododendron aureum* and its coexisting species

	Chi‐square	*df*	A	*r*
*Vaccinium* spp.	66.80	9	0.64	−.22
*Rhododendron bracteatum*	73.03	3	0.66	−.22
*Dryas octopetala*	121.43	6	0.75	−.25
*Rhododendron confertissimum*	111.46	3	0.73	−.15
*Phyllodoce caerulea*	102.03	3	0.72	1.00
*Juniperus sibirica*	78.63	4	0.67	−.06
*Salix divaricata*	96.80	3	0.71	−.47
*Vaccinium vitis*	76.97	4	0.67	−.60

RDA was used to investigate the impact of plant community structure (Figure 7b). The results show that elevation, MBC, and MBN significantly correlate with community composition (Table S5). Many of the dominant species positively correlate with each other (Figure 7b), thereby contributing to the overall interspecific association observed; however, *R. aureum* negatively correlates with half of the dominant species, including both shrub and herbaceous species (Figure 7b). This suggests that *R. aureum* either competes for resources or interferes with other species.

## 4 DISCUSSION

A species can survive and become widely distributed in an alpine environment not only based on its ability to adapt to abiotic stresses, but also its ability to cope with biotic influences. Both competition and facilitation must be considered in order to obtain a realistic view of the interactions within alpine plant communities (Kikvidze, 2002; Olofsson, 2004). The overall interspecific association among dominant shrubs on the Changbai Mountain exhibits a significant positive association. Many studies propose the stress gradient hypothesis, in which plants exhibit positive relationships with increasing stress in alpine ecosystems, especially in high altitude areas (Callaway et al., 2002; Choler, Michalet, & Callaway, 2001; Reid, Lamarque, & Lortie, 2010). Changbai Mountain is a typical alpine environment with low temperatures, heavy precipitation, short‐growing seasons, strong winds, high solar radiation, and low resource availability. Facilitation appears to be driven by architecture‐mediated protection from winds and low temperatures, as well as avoiding resource competition (Baumeister & Callaway, 2006). Nevertheless, *R. aureum* shows significant negative associations with most dominant shrubs and herbs, suggesting interspecific competition. A negative relationship between species can occur when resource abundance drives abiotic stress (Kikvidze et al., 2005; Maestre et al., 2009), especially in the infertile alpine soil.

In this study, we first examined whether the *R. aureum* plant community can form its own unique soil environment, which is distinct from other plant communities. A deeper understanding of the effects of vegetation types on the soil microbiome in alpine ecosystems can help to develop better strategies to protect the harsh environment of the alpine tundra (Klimeš, 2006). We started with the hypothesis that *R. aureum* supports a unique soil system allowing for its expansion and succession within the subalpine ecosystem; however, contrary to this, no significant difference was seen for most soil nutrients between sites with and without *R. aureum*. Many of the soil properties significantly correlated with elevation, which is consistent with previous studies (Wu et al., 2015). This suggests that elevation strongly influences soil properties, such as total nitrogen, organic matter, and some enzymatic activities, which decrease as elevation increases. Some soil properties were significantly different between the vegetation types in this study. For instance, soil microbial biomass (MBN and MBC), urease, and sucrase were higher at sites with *R. aureum*. A large amount of organic carbon (C) conversion, storage, and respiration by microorganisms in the soil (Spohn, 2015), and in soil microbial biomass, is an important determinant for soil carbon dynamics (Thakur et al., 2015). Thakur et al. (2015) suggested that higher plant diversity has a strong effect on soil microbial communities and could increase microbial biomass. Zak, Holmes, White, Peacock, and Tilman (2003) also revealed that, within a given region, microbial biomass increases with plant productivity. Furthermore, recent studies have highlighted that higher soil microbial biomass is driven by an input of organic matter, nitrogen, and phosphorus, as well as regulation of soil moisture (Lange et al., 2015; Liu et al., 2015; Thakur et al., 2015). Greater total nutrients were observed in soils with *R. aureum* than in soils without *R. aureum*, indicating that plant community establishment plays a crucial role in microbial biomass accumulation.

A greater abundance of nitrogen‐fixing bacteria, extracellular enzymes, and nitrogen‐cycling functional groups in soils with *R. aureum* could increase nutrient availability. Nitrogen is one of the most important limiting factors for organism productivity; thus, nitrogen‐fixing organisms play a key role in microbial and plant biomass production, as well as organic matter accumulation in soils (Jhp et al., 1997). Nitrogen‐fixing bacteria are important functional microorganisms in soil quality (Ahmad et al., 2016) because they promote the establishment and growth of plants by increasing the nutrient supply (Jasper, Abbott, & Robson, 1989; Liang, Pan, He, Chen, & Su, 2016). Our results show that the abundance of N‐fixing genes has a significant positive correlation with soil nutrients, especially total nitrogen and available nitrogen. This is consistent with our hypothesis that the *R. aureum*‐associated soil harbors microbes that provide nutrition to promote *R. aureum* growth.

Soil pathogens have distinct spatial and temporal patterns within the natural plant community (Van der Putten, 2003). Plant succession can also be driven by soil pathogens when the accumulation of pathogens leads to the extinction of the dominant plant and replacement with other plant species that are immune or insensitive to the pathogens (Putten, Dijk, & Peters, 1993). *R. aureum* is relatively pathogen tolerant, as demonstrated by the higher abundance of pathogens and lower abundance of antibiotic‐producing bacteria in its associated soil. The observed pathogens would likely arrest the growth of other species that are sensitive to them. Due to the competitive relationship between *R. aureum* and other species, the higher relative abundance of pathogens may threaten the survival of other species, so as to reduce species diversity. In the future, it is necessary to pay attention to the influence of *R. aureum* growth on the plant diversity in alpine tundra.

In‐depth analysis showed that the microbial community in soils with *R. aureum* had lower diversity than soils without *R. aureum*; however, the difference was not significant, even though there was a difference between the microbial community structures in the different vegetative soils. Moreover, NMDS ordination revealed that the soil microbial structure was distinct between sites with and without *R. aureum*. The heatmap showing pairwise comparisons of the bacterial community structures between all samples agrees with the NMDS results. Furthermore, based on the results of the RDA (Figure 6a), the bacterial community composition was significantly affected by soil pH and total nitrogen. Correlation analysis indicates a significant positive relationship between soil pH and the three dominant microbial phyla (Gemmatimonadetes, Bacteroidetes, and Nitrospirae). Furthermore, total nitrogen significantly correlated with two of the dominant microbial phyla (Gemmatimonadetes and Fusobacteria). Soil bacteria are more sensitive to factors such as pH, moisture, nutrient availability, and C/N ratios (Liu et al., 2014; Uroz, Tech, Sawaya, Frey‐Klett, & Leveau, 2014). Shen et al. (2013) suggested that the bacterial community structure in Changbai Mountain soils at different altitudes is mainly affected by pH. Different plant types have different effects on the soil (Wu et al., 2015; Zhang, Liu, Xue, & Xiao, 2013), and soil microbial community structure over altitudinal gradients is mainly governed by changes in the vegetation cover type (Singh et al., 2014).

Plants can change the physical, chemical, and biological characteristics of the surrounding soil environment, thereby affecting the future growth of plants in that area. In order to study the interaction between plant community structure and soil properties, RDA was used to demonstrate that microbial biomass (MBN and MBC) and elevation significantly affected plant community structure (Figure 6b). Many past studies have shown that microbial biomass contributes to plant productivity; however, our research provides evidence that it also affects the structure of the plant community. In this analysis, not all soil properties significantly affect the plant community, which may be related to plant growth characteristics. The vertical distribution of vegetation in Changbai Mountain is known to mirror the horizontal vegetation zonation from temperate to frigid zones on the Eurasian continent (Xu, He, Chen, & Liu, 2004). This study also shows that elevation drives the spatial distribution of plant communities on the Changbai Mountain.

In summary, we demonstrate that the nitrogen‐fixing and pathogenic bacteria are more abundant in soil with an *R. aureum* community than in soils without this community. Despite the lack of significant variation in microbial diversity, the microbial communities in the two groups could be clearly divided into two clusters. Therefore, we conclude that *R. aureum* facilitates a unique soil microbial community structure, which is distinct from other plant species. The plant community structure significantly correlates with elevation, as well as microbial biomass carbon and nitrogen. Elevation may drive the spatial distribution of plant communities on Changbai Mountain. In addition, *R. aureum* exhibits a negative association with most other dominant shrubs and herbs, suggesting interspecific competition.

## CONFLICT OF INTEREST

None declared.

## AUTHORS CONTRIBUTION

W.X.L. and L.L. conceived the study. L.L., W.X.L., Z.W., and Z.J.X. performed the experiments. W.X.L. and C.X. interpreted the results and W.X.L. wrote the manuscript.
