# Supplementary material for: Rhododendron aureum Georgi formed a special soil microbial community and competed with above‐ground plants on the tundra of the Changbai Mountain, China
Source: Ecol Evol. 2017 Aug 11;7(18):7503–14. doi: 10.1002/ece3.3307 (PMC5606866; doi:10.1002/ece3.3307)
Supplement: Supplementary file 1 [file ECE3-7-7503-s001.docx]

***Rhododendron aureum* Georgi formed a special soil microbial community and competed with above ground plants on the tundra of the Changbai Mountain, China**

**Xiaolong Wang ^1^, Lin Li ^1^, Wei Zhao ^1^, Jiaxin Zhao ^1^, Xia Chen ^1*^**

1.National & Local United Engineering Laboratory for Chinese Herbal Medicine Breeding and Cultivation, School of Life Sciences, Jilin University, Changchun 130112, China.

^*^For correspondence. E-mail address: [chenxiajlu@163.com](mailto:chenxiajlu@163.com)

Key words: microbial biomass, interspecific competition, microbial community, function microbial

TableS1. Primers and real-time PCR conditions used in this experiment.

| Function genes | Primers | | Real-time PCR protocol |
| --- | --- | --- | --- |
| *nifH* | PolF | 5′-TG CGAY CCS AAR GCB GAC TC-3′ | 94°C for 5 min; 40 cycles of 94°C for 30s, 57°C for 45s(54°C for *nifH* gene, 60°C for AOB), 72°C for 1 min. |
|  | PolR | 5′-ATS GCC ATC ATY TCR CCG GA-3′ |  |
| AOA | *Arch-amoA*F | 5′-STA ATG GTC TGG CTT AGA CG-3′ |  |
|  | *Arch-amoA*R | 5′-GCG GCC ATC CAT CTG TAT GT-3′ |  |
| AOB | *amoA*-1F | 5′-GGG GTT TCT ACT GGT GGT-3′ |  |
|  | *amoA*-2R | 5′-CCC CTC KGS AAA GCC TTC TTC-3′ |  |
| *nosZ* | *nosZ*-F | 5′-CGY TGT TCM TCG ACA GCC AG-3′ |  |
|  | *nosZ*-1622R | 5′-CGS ACC TTS TTG CCS TYG CG-3′ |  |

Table S2 Pearson correlation coefficient (r) between soil properties elevation and elevational, Bolded values indicate contrasts that are significantly different (P ≤ 0.05) between different treatments.

|  | TN | AN | TP | AP | TK | AK | NO_3_^-^ | NH_4_^+^ | TOC | pH | MBC | MBN | Moisture |
| --- | --- | --- | --- | --- | --- | --- | --- | --- | --- | --- | --- | --- | --- |
| *R* | -0.69 | -0.75 | -0.10 | -0.40 | 0.68 | -0.53 | 0.43 | 0.32 | -0.68 | 0.66 | -0.54 | -0.50 | -0.62 |
| *P*-value | **0.00** | **0.00** | 0.72 | 0.12 | **0.00** | **0.03** | 0.10 | 0.23 | **0.00** | **0.01** | **0.03** | **0.05** | **0.01** |

Table S3 Alpha Diversity Metrics for *R. aureum* sites and other species *R. aureum* without bacteria.

|  | chao1 | Shannon index | OTU richness |
| --- | --- | --- | --- |
| *R. aureum* | 980.15 | 7.55 | 826.00 |
| *X* | 1094.03 | 7.77 | 900.00 |
| *P*-value | 0.40 | 0.29 | 0.38 |

Table S4 The correlation between community composition and environmental variables for bacteria. Values in bold indicate significant correlation (*P*<0.05).

|  | *r^2^* | *Pr*(>r) |
| --- | --- | --- |
| Altitude | 0.32 | 0.09 |
| TN | 0.43 | **0.02** |
| AN | 0.22 | 0.24 |
| TP | 0.29 | 0.12 |
| AP | 0.32 | 0.09 |
| TK | 0.30 | 0.12 |
| AK | 0.33 | 0.10 |
| NO_3_^-^ | 0.29 | 0.11 |
| NH_4_^+^ | 0.03 | 0.81 |
| TOC | 0.20 | 0.31 |
| pH | 0.54 | **0.03** |
| MBC | 0.22 | 0.21 |
| MBN | 0.15 | 0.31 |
| Moisture | 0.29 | 0.12 |

Table S5 The correlation between community composition and environmental variables for plant. Values in bold indicate significant correlation (*P*<0.05)

|  | *r^2^* | *Pr*(>r) |
| --- | --- | --- |
| Altitude | 0.40 | **0.03** |
| TN | 0.12 | 0.43 |
| AN | 0.22 | 0.22 |
| TP | 0.18 | 0.30 |
| AP | 0.02 | 0.87 |
| TK | 0.15 | 0.37 |
| AK | 0.11 | 0.40 |
| NO_3_^-^ | 0.12 | 0.42 |
| NH_4_^+^ | 0.18 | 0.31 |
| TOC | 0.13 | 0.41 |
| pH | 0.34 | 0.07 |
| MBC | 0.43 | **0.04** |
| MBN | 0.36 | **0.02** |
| Moisture | 0.30 | 0.10 |


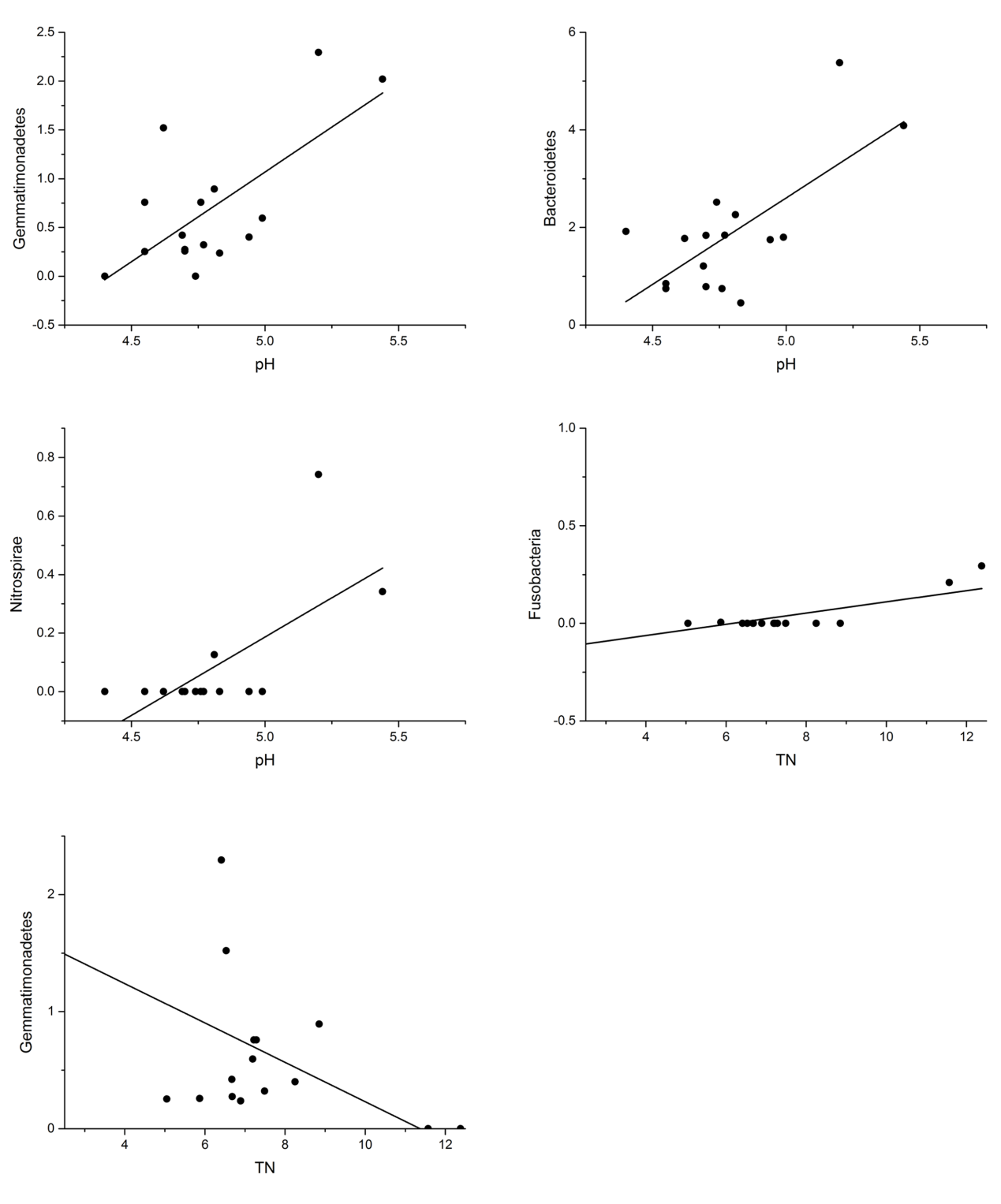


Fig.S1 The relative abundance of dominant microbial was correlation with pH and TN (P<0.05).
